# In situ monitoring of photocatalyzed isomerization reactions on a microchip flow reactor by IR-MALDI ion mobility spectrometry

**DOI:** 10.1007/s00216-020-02923-y

**Published:** 2020-09-12

**Authors:** Chris Prüfert, Raphael David Urban, Tillmann Georg Fischer, José Villatoro, Daniel Riebe, Toralf Beitz, Detlev Belder, Kirsten Zeitler, Hans-Gerd Löhmannsröben

**Affiliations:** 1 University of Potsdam, Physical Chemistry, Karl-Liebknecht-Str. 24-25, 14476 Potsdam, Germany; 2 Institute of Analytical Chemistry, Leipzig University, Linnéstraße 3, 04103 Leipzig, Germany; 3 Institute of Organic Chemistry, Leipzig University, Johannisallee 29, 04103 Leipzig, Germany

**Keywords:** Microchip, Reaction monitoring, IR-MALDI, Ion mobility spectrometry, Photochemistry, Photocatalysis, Olefin isomerization

## Abstract

The visible-light photocatalytic *E*/*Z* isomerization of olefins can be mediated by a wide spectrum of triplet sensitizers (photocatalysts). However, the search for the most efficient photocatalysts through screenings in photo batch reactors is material and time consuming. Capillary and microchip flow reactors can accelerate this screening process. Combined with a fast analytical technique for isomer differentiation, these reactors can enable high-throughput analyses. Ion mobility (IM) spectrometry is a cost-effective technique that allows simple isomer separation and detection on the millisecond timescale. This work introduces a hyphenation method consisting of a microchip reactor and an infrared matrix-assisted laser desorption ionization (IR-MALDI) ion mobility spectrometer that has the potential for high-throughput analysis. The photocatalyzed *E*/*Z* isomerization of ethyl-3-(pyridine-3-yl)but-2-enoate (***E*-1**) as a model substrate was chosen to demonstrate the capability of this device. Classic organic triplet sensitizers as well as Ru-, Ir-, and Cu-based complexes were tested as catalysts. The ionization efficiency of the *Z*-isomer is much higher at atmospheric pressure which is due to a higher proton affinity. In order to suppress proton transfer reactions by limiting the number of collisions, an IM spectrometer working at reduced pressure (max. 100 mbar) was employed. This design reduced charge transfer reactions and allowed the quantitative determination of the reaction yield in real time. Among 14 catalysts tested, four catalysts could be determined as efficient sensitizers for the *E*/*Z* isomerization of ethyl cinnamate derivative ***E*-1**. Conversion rates of up to 80% were achieved in irradiation time sequences of 10 up to 180 s. With respect to current studies found in the literature, this reduces the acquisition times from several hours to only a few minutes per scan.

**Electronic supplementary material:**

The online version of this article (10.1007/s00216-020-02923-y) contains supplementary material, which is available to authorized users.

## Introduction

Preparative photoreactions carried out in stirred batch reactors are regularly characterized by reaction times on the scale of hours. This is due to the usually high optical density of the reaction solutions and the resulting low penetration depth of light. In order to achieve low and well-defined liquid layer thicknesses, a number of photoreactor designs, such as flatbed, falling film, capillary flow, and microchip reactors, were developed. Especially microchip reactors have become popular in recent years.

The narrow width of the tubing in capillary flow reactors enables uniform irradiation of the reaction volume. This also offers a series of advantages over photochemistry in batch photoreactors, such as an accurate control over reaction parameters, or the improved heat transfer due to the larger surface-to-volume ratio. Moreover, there is a controlled mixing of fluids, an enhanced reactivity due to the increased photon flux in photochemical reactions, and continuous multistep syntheses are possible [1]. Additionally, the product is constantly spatially separated from the irradiated reaction zone, which reduces the degree of photodegradation of products and increases the productivity of photochemical processes [2, 3]. An overview of the photoreactions in capillary flow reactors can be found in [1, 2, 4, 5].

While chip-based reactors benefit from similar advantages as capillary flow reactors, they are much more compact and therefore allow enhanced integration of various functionalities on a single device. In addition, chip-based devices can mix solutions dead-volume-free and different reaction zones on the chip containing different solvents can be introduced. The latter enables decoupling of different flow rates in different channels for reaction progression and subsequent analysis [6]. Furthermore, the combination of chemical reactions and online analysis enables time resolved studies of chemical processes. The latter can be realized by applying spectroscopic techniques, such as fluorescence or Raman microscopy, or by coupling microfluidic chips to mass spectrometry. For isomer discrimination, we previously reported the integration of microflow reactions and chip HPLC [7–9] that allowed for the analysis of enantioselective transformations.

Visible light is an inexhaustible source of energy to drive “green” chemical processes. Photocatalysts can be useful as photosensitizers that can absorb visible light and drive reactions of organic molecules via electron or energy transfer, which could otherwise only be carried out using UV light. The absorption of visible light allows new modes of molecule activation and enables challenging or previously unattainable transformations. Iridium or ruthenium polypyridyl complexes are among the most applied photocatalysts. By now, a broad range of novel synthetic methods became accessible through visible-light photocatalysis [10, 11]. In recent years, the direct application of solar radiation for performing photocatalyzed syntheses has also been explored [12–14].

Gilmour et al. investigated that the photocatalytic, bio-inspired *E/Z* isomerization of olefins yields almost pure *Z*-isomer (up to 99:1 *Z*/*E*) by using different photocatalysts, in batch processes over 24 h [15]. This isomerization is initiated by a triplet energy transfer from the photocatalyst to an α,β-unsaturated ester and was studied for a series of α,β-unsaturated esters and various photocatalysts, which possess triplet energies in the range of 140 to 250 kJ/mol [15].

A variety of spectroscopic methods have been implemented in order to monitor reactions performed on microfluidic chips. A waveguide interferometer–based UV-Vis spectrometer was used for the detection of an antibody-antigen reaction [16, 17]. However, UV-Vis absorption spectroscopy has a rather poor sensitivity due to the short optical path lengths found in microfluidic chips. An alternative method is fluorescence spectroscopy, which was utilized for the analysis of reaction progress [18] after chromatographic or electrophoretic separation [19–21]. A third method is surface-enhanced Raman spectroscopy, the suitability of which was demonstrated through several studies: by monitoring the progress of a Hantzsch synthesis in droplets [22], in a kinetic study of a platinum-catalyzed reduction of 4-nitrothiophenol to 4-aminothiophenol [23], monitoring the Fenton degradation of rhodamine 6G [24] and the products of a multistep cascade reaction of glycerol to mesoxalic acid [25].

In addition to optical methods, mass spectrometry (MS) allows tracing chemical reactions on the microchip in real time. An example is the monitoring of stereoselective transformations on a microchip by chip-integrated HPLC separation and subsequent electrospray ionization mass spectrometry detection (ESI-MS) [7]. Alternative to ESI, infrared microbeam matrix-assisted laser desorption and ionization (IR-MALDI in high vacuum) MS can be employed. This methodology was used to investigate the kinetics of the synthesis of pyrrolo[2,1-b]benzoxazoles [3]. IR-MALDI is a very soft ionization method that allows detection of short-lived and sensitive intermediates. In the MS configuration applied in [3], the microbeam was situated in the high vacuum region of the MS, enabling the direct transfer of the intermediates into that region after IR-MALDI and thus their immediate detection. IR-MALDI-MS, at atmospheric pressure (AP), has been used to investigate a vinylogous Mannich reaction performed in continuous flow on a microchip [6]. Special features of the microchip were the on-chip dilution step with an IR-MALDI compatible solvent and the chip-integrated nozzle to generate the microbeam.

Ion mobility (IM) spectrometry is a fascinating alternative to MS. In contrast to the time-, cost-, and labor-intensive high-performance liquid chromatography-MS, IM spectrometry is an alternative method with the potential to separate and detect isomeric compounds. This has already been demonstrated for the separation of isomers produced by photoreactions in the gas phase [26, 27] and the photoisomerization of azobenzenes [28]. Analogous to MS, the composition of the reaction mixture in flow reactors can be determined in real time but at reduced costs. The combination of these properties makes IM spectrometry attractive for reaction monitoring. IM spectrometry in combination with ESI was previously used for reaction monitoring of a three-step synthesis [29]. The coupling of a microchip-based HPLC separation and IM spectrometry was also shown [30], demonstrating the potential of chip-HPLC/IM spectrometry as a miniaturized two-dimensional separation method for separating three isomeric antidepressants.

In this work, the photocatalyzed *E*/*Z* isomerization of the ethyl-3-(pyridine-3-yl)but-2-enoate (***E*-1)** in photo flow reactors was investigated by IR-MALDI-IM spectrometry. This technique allows the real-time separation and detection of the *E-* and *Z*-isomer. Two different photo flow reactors, a capillary and a microchip reactor, were studied. Capillary reactors have the advantage of easier handling due to their modular design but were usually only partially illuminated by the intense irradiation of a single LED. Microchip reactors allow for integrated reaction zones and hence to effectively realize complex mixing processes. Since charge transfer processes at atmospheric pressure can influence the detection of the reaction products, a low-pressure IM spectrometer was used. Herein, we demonstrated the capability of this hyphenated method of microchip and IM spectrometer for high-throughput analysis of a photochemical reaction. Moreover, the effects on the reaction yields of the *E*/*Z* isomerization of a number of photocatalysts were investigated.

## Experimental part

### General instrumentation and method

**Fig. 1:**
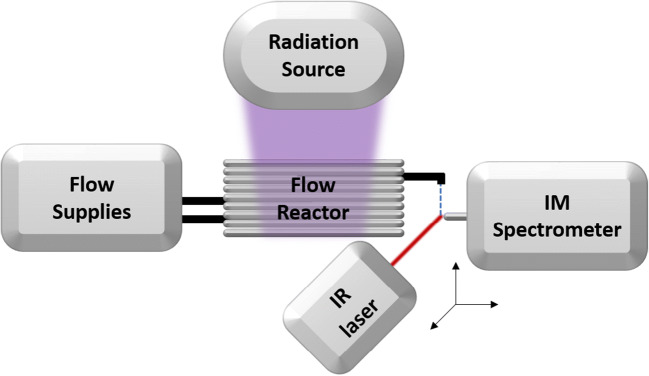
Scheme of the general setup consisting of the liquid flow supply unit, the flow reactor unit, a radiation source, the ion mobility (IM) spectrometer unit, and an OPO IR laser

The experimental setup consisted of the IR laser, the liquid flow supply, the flow reactor, the radiation source, and the IM spectrometer and is schematically shown in Fig. 1. Two different flow reactors were used: a microchip reactor and a capillary flow reactor as reference.

In brief, the *E*-isomer ***E*-1** (2.5 mM) and the respective catalyst (2 mol%) were dissolved in MeCN:MeOH 1:1 (v/v) and irradiated with a single LED (*λ* = 404 ± 15 nm or 365 ± 15 nm) for 10 s in the capillary or 180 s on the microchip. The reaction mixture was diluted (1:99) on-chip and then analyzed by an IR-MALDI-IM spectrometer built in-house. Measurements were performed as follows: a sample of the starting material solution was injected at *t* = 0 s. After the fluid passed through the microchip or the capillary reactor and a stable signal for the starting material was observed, the LED was turned on. Depending on the photocatalyst, the *Z*-isomer was formed as a product and was detected by the IM spectrometer. After a full residence time cycle, the LED was switched off and the measurement was stopped. Then, only starting material signal was observed.

### Liquid flow supply

Two HPLC pumps (Infinity 1260, Agilent and Azura P 6.1 L, Knauer, GER), an injection valve, and a UV-transparent fused-silica capillary (0.91 m, ID 75 μm, FS UV untreated, CS-Chromatographie, GER) made up the microfluidic system for the first setup. In a second setup, the UV-transparent capillary was replaced by a microchip, providing a tightly meandering reaction channel that further enhances the irradiation efficiency. At the end of the photo flow reactors, a make-up flow was added that dilutes the reaction mixture (1:99) and establishes conditions compatible with IR-MALDI. The make-up flow rates were varied between 79 and 396 μL/min depending on the used reaction channel flow rate. The dilution factor was kept constant.

The solvent, provided by the two HPLC pumps, was delivered through polyether ether ketone (PEEK) capillaries (OD 1/16″, ID 50 μm, JR-T-5802-M5, VICI AG International, CH) into the injection valve. In the capillary reactor setup, a 6-port valve (Azura V 2.1, Knauer, GER) with a 20-μL sample loop was used, and in the microchip reactor setup, a 10-port valve (C72MPKH-4670ED, VICI AG International, CH) with a 50-μL sample loop was used. The reaction mixture was introduced into the sample loop via microinjection ports (M-432-03, IDEX Health & Science, USA) and through in-line filters (JR-0611-SS05-3, VICI AG International, CH) protecting the reaction channels from clogging. For fluid introduction into the microchip, the tubing was connected to polyacrylamide-coated fused-silica capillaries (FS, OD 360 μm, ID 50 μm, Chromatographie Service GmbH, GER) by reducing adapter assemblies (V-447, Upchurch Scientific, IDEX Health & Science, USA). To ensure a pressure-tight interface between the microchip and the fused-silica capillaries, in-house-built stainless-steel connection clamps consisting of perfluoro-elastomeric (FFKM) ferrules and headless 6–32 PEEK screws (N-123-04 and N-123H, IDEX Health & Science, USA), were used [31].

### Microchip flow reactor

The microfluidic glass chips were designed and fabricated in-house by common photolithography, wet-etching, and bonding methods [32]. A description is found in the Electronic Supplementary Material (ESM). Briefly, the microchip consisted of two layers, both of which were manufactured from microscope slides of a soda-lime glass with a size of 76 mm × 26 mm (Carl Roth GmbH + Co. KG, GER). The bottom glass-layer contained etched channels with a depth of 20 μm and a width of 100 μm. To close the channels, the slide was bonded to a top layer with powder-blasted holes that served as inlets for pressure-tight fluid delivery. The glass microchip contained the following functional units: The channel (50 nL) led to a reactor region with an internal volume of 560 nL. After the reactor region, the channel (70 nL) crossed a make-up flow channel where additional fluid (MeCN:H_2_O 1:1 (v/v), 1:79 up to 1:299 dilution of the reaction mixture) was delivered to form a liquid jet at the end of the microchip. A pulled glass emitter tip was manufactured at the microchip exit to reduce the flow rate necessary to generate a free-standing liquid jet [33, 34]. The channel had a total internal volume of approximately 680 nL. This channel then tapered into a 20-μm opening which generated the free-standing liquid jet of about 20 μm diameter.

### Capillary flow reactor

The in-house synthesized *E*-isomer ***E*-1** was dissolved in MeCN (5.0 mM) and mixed with a 100 μM (2 mol%) solution of the respective catalyst in MeCN right before analysis in a 1:1 volumetric ratio. The sample was injected into a 20-μL sample loop of the 6-port valve. From here, the reaction mixture was picked up by a stream of MeCN of usually (0.3–3.0) μL/min and was transferred into a reaction capillary loop (*l* = 0.90 m, *V* = 1.8 μL) with three windings. When the thrice-turned UV-capillary was supplied with 1 μL/min flow rate of analyte solution, a linear velocity of 3.8 mm/s and thus a Reynolds number of about 2 were achieved, indicating laminar flow conditions. Each tube winding has a length of approximately 30 cm. Since the LED had an irradiating surface area of about 1 cm^2^, only a part of the total capillary volume was intensely irradiated. This means that the reaction mixture was irradiated three times for a total time of about 10 s, depending on the exact sample flow rate. After that, the reaction mixture was diluted into a make-up flow of MeCN:H_2_O 1:1 (v/v) giving a total flow rate of 300 μL/min. This terminated the reaction progress at a defined point in time and generated sufficient throughput to create a free-standing liquid beam of 20 μm in diameter by means of a glass nozzle (OD 1.0 mm, ID 0.235 mm, tapered to ID = 20 ± 2 μm at the nozzle tip, Biomedical Instruments, GER).

### Radiation sources

For conducting the photoreactions on microchip, a light-emitting diode (LED, 404 nm, operated at 9 A, 3.55 V; CUN0MF9A, Seoul Viosys, KR) was installed in front of the reactor region at a distance of 3 mm and was cooled at the backside by an axial fan (HXB25B12, SEPA EUROPE, GER). Alternatively, a 365-nm LED was used, which had about 2% of the intensity of the 404-nm LED.

### IR-MALDI-IM spectrometer

**Fig. 2:**
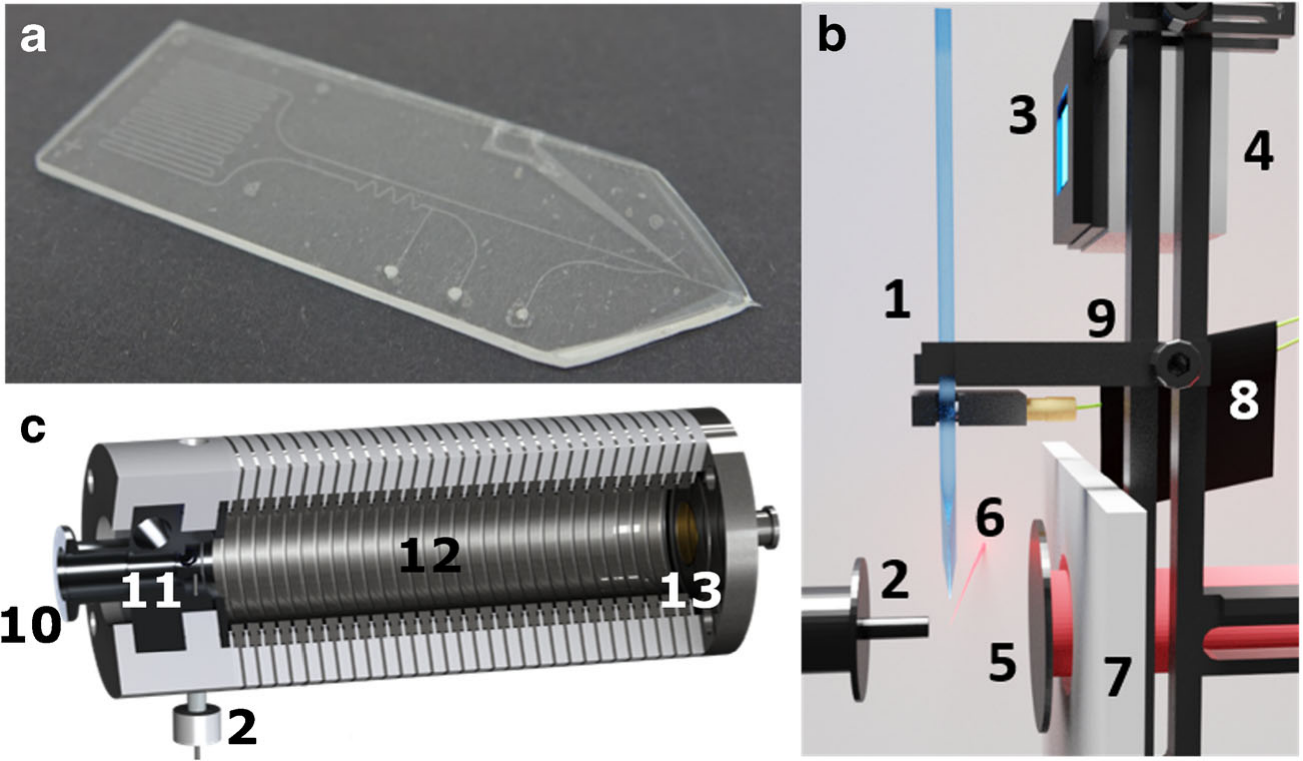
**a** Microchip used in this study. **b** Layout of chip setup in front of IM spectrometer inlet with a side view of the microfluidic chip (1), the IM spectrometer inlet (2), the LED positioned in front of the chip reactor region (3), the LED cooler (4), the counter electrode (5), the laser beam (6), a PTFE insulation plate (7), the capillary system and microchip mount including clamps (8), and a fine positioning stage (9). **c** In-house-built IR-MALDI IM spectrometer with heated inlet in top view, inlet (2) at the bottom-left is equivalent to (2) of panel **b**, the pump inlet (10), the pulsed ion inlet (11), the drift tube (12), and the faraday plate (13) as the detection element

The detection of the isomers is based on an in-house-built IM spectrometer as described in a previous publication [35]. A liquid filament, called microbeam, at an atmospheric pressure was used for coupling the photo microchip or the photo capillary reactor to the reduced pressure IM spectrometer [36]. The microchip is mounted between a counter electrode and the inlet electrode of the IM spectrometer (Fig. 2a). To support the liquid beam and to guide charged analytes into the orifice by an electric field (next to the pressure gradient), a counter electrode at a voltage of 7 kV was positioned at the opposite side of the inlet electrode and the liquid beam. This geometry ensured the most uniform electric field around the ionization region. Under nominal operating conditions, the counter electrode was set at a distance of 4 mm from the inlet electrode with the microchip outlet placed midway between them. The laser focal point and the microchip outlet were positioned at a distance of 2 mm in front and 2 mm above of the IM spectrometer orifice with the help of an *X*-*Y*-*Z* linear translation stage (Metric XYZ Three-Axis Miniature Translation Stage, Thorlabs GmbH, GER). Throughout all experiments, the capillary and the optical systems were fixed in the laboratory frame to keep the laser beam path’s focal point unaltered.

A nanosecond IR laser pulse (*λ* = 2940 nm, rep. rate = 20 Hz, *t* = 6 ns, *E* = 2.1 ± 0.1 mJ, IR Opolette 2940, OPOTEK, USA) was guided through a 99 ± 0.5-cm-long optical pathway achieved with two gold mirrors (PF10-03-M01, Thorlabs GmbH, GER) and a CaF_2_ plano-convex lens (*f* = 75 mm, Edmund Optics GmbH, GER) that focused the laser beam onto the microbeam.

In order to monitor the ionization region by stroboscopic imaging, a CCD camera (DBK 41 AU02, The Imaging Source, GER) was used. On the opposite side of the ionization region, a red stroboscopic diode (LED and electronics by Autodrop AD-K-901, microdrop Technologies GmbH, GER) was mounted within the optical axis of the CCD camera. The diode power supply unit was triggered by the OPO laser Q-switch output signal via a delay generator (PDG 204, S.M.V. München, GER) to correct for any optical and electrical delays and to observe different points in time before and after the laser impact on the free-standing liquid beam.

The IR laser dispersed a volume of the liquid beam, generating analyte ions. The ionized analytes entered the heated transfer tube, where desolvation took place. The ions were then pulsed into the IM spectrometer for 140–340 μs (pulse width), where they were separated according to their size and shape and detected by a Faraday plate (20 mm diameter), coupled to an amplifier (1 GV/A, custom-built by ISAS, GER) and then fed to an oscilloscope (TDS 5052 PDO, Tektronix Inc., USA) for data acquisition.

The heated transfer tube (200 °C) bridges the different pressure regimes: the ambient pressure ionization region and the reduced pressure IM spectrometer (100 mbar N_2_, 0.3 L/min N_2_, 60 °C, 212 mm drift tube, 140–340 μs pulse width, and 300–540 μs pulse delay). After the laser pulse was released, the laser triggered the electric potential switch of the ion pulsing via a delay generator and a fast high-voltage switch (GHTS 30, Behlke Power Electronics GmbH, GER). The system was usually operated with a pulse delay of 310 μs, a pulse width of 140 μs, and a repetition rate of 20 Hz. This resulted in a resolving power (defined as drift time over full width at half maximum) of 20–40 for the system, which is sufficient to separate the two isomers well enough without compromising the sensitivity. It also allows the calculation of the yield for each species by a simple cumulative Gaussian fit.

## Results

### Photoreaction

An example of a visible light–catalyzed photoreaction is the isomerization of the *E*-isomer, ***E*-1**, to the corresponding *Z*-isomer ***Z*-1** by photocatalysts (see reaction scheme 1). After excitation of the photocatalyst by visible light, the reaction, based on triplet energy transfer, can occur yielding the *Z*-isomer ***Z*-1**. After breaking the π bond, the energy transfer leads to the formation of a delocalized biradical electron system [15] as an intermediate that allows the C-C bond to rotate. The discrimination of both isomers is based on the deconjugation of the π system of the *Z*-isomer, in which the side chain is sterically rotated out of the plane of the aromatic system. This increases the triplet energy of the *Z*-isomer and renders triplet energy transfer from the catalyst to the *Z*-isomer. This makes re-isomerization of the *E*-isomer infeasible.1

In addition to photocatalysts such as riboflavin (**L**), a number of other photocatalysts for which this isomerization has already been observed, were also investigated in this work [15]. A summary is given in Table 1.

**Table 1:** Overview of photocatalysts investigated

Name	Label	*E*_TRIPLET_ (kJ/mol)
[Ru(bpy)_3_](PF_6_)_2_	A	195 [10]
[Ru(phen)_3_] Cl_2_	B	196 [10]
Rose Bengal	C	198 [15]
[Ru(bpz)_3_](PF_6_)_2_	D	201*
Mes-Arc^+^ClO_4_^−^	E	205 [37]
9-Fluorenone	F	209 [38]
Eosin Y	G	210 [39]
[Ir(dtbbpy)(ppy)_2_]PF_6_	H	212 [40]
Rhodamine 6G	I	218 [41]
4CzIPN	J	220*
*fac*-Ir(ppy)_3_	K	232 [10]
Riboflavin	L	234*
[Ir[dF(CF_3_)ppy]_2_(dtbbpy)]PF_6_	M	252*
[Cu(dap)_2_]Cl	N	160 [42]

*Experimental data can be found in the ESM

### ESI/IR-MALDI IM detection of *E*/*Z* isomers

**Fig. 3:**
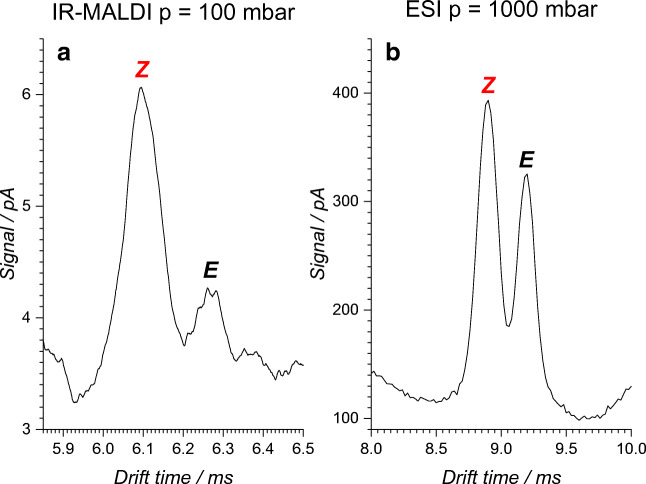
Comparison of IM spectra of 50:50 mol% *E*/*Z* mixtures of an ethyl cinnamate derivative ***E*-1**: **a** IR-MALDI-IM spectrum at 100 mbar and **b** ESI-IM spectrum at an ambient pressure. Differences in relative and absolute intensity of the isomers arise from the different charge transfer equilibria for the different techniques and pressures. In both cases, a good separation between the *E-* and the *Z*-isomer was achieved

The advantage of drift tube IM spectrometry, compared with MS, is the facile differentiation of isomeric compounds, and the reduced measuring time compared with chromatographic methods. Similar to MS, the composition of the reaction mixture in flow reactors can be determined in real time. The combination of both properties, the differentiation of isomeric compounds and the real-time analysis, makes drift tube IM spectrometry attractive for reaction monitoring. The ionization of the components of the reaction mixture should be carried out gently. If polar or aqueous solvents are used, IR-MALDI can be applied alternatively to ESI. As demonstrated in Fig. 3, the *E*- and *Z*-isomers of ethyl cinnamate derivative ***E*-1** and ***Z*-1** were successfully ionized by both methods. The IM spectra also show that both isomers were well separated in the IM spectrometer. An IM spectrometer working at AP was used to obtain the ESI-IM spectra. In the remainder of this work, IR-MALDI was used as the ionization method. IR-MALDI has distinct advantages over ESI. These include the formation of ions that are usually only singly or doubly charged, the softer ionization of the analytes, for example, for electrochemically reactive substances, and the higher sensitivity in combination with pulsed analysis methods (time-of-flight). Another important contribution to this work is the ability of IR-MALDI to tolerate high water contents and wide ranges of flow rates. The flow rate within the reaction channel on the microchip was typically 0.3–1 μL/min and within the flow reactor 1–3 μL/min, so that a high reaction rate was realized. At the microchip and capillary reactor outlet, the liquid stream of the reaction mixture was mixed with a liquid stream of a MeCN/H_2_O mixture (100 μL/min and 300 μL/min). This stopped the reaction, diluted the reaction mixture, and achieved an optimal solvent composition for subsequent analysis. While the resulting flow rate was optimal for IR-MALDI because it created a microbeam which was sampled by the IR-laser, this flow rate would have been too high for the combination of ESI and the IM spectrometer.

### Monitoring of the photocatalyzed *E*/*Z* isomerization

**Fig. 4:**
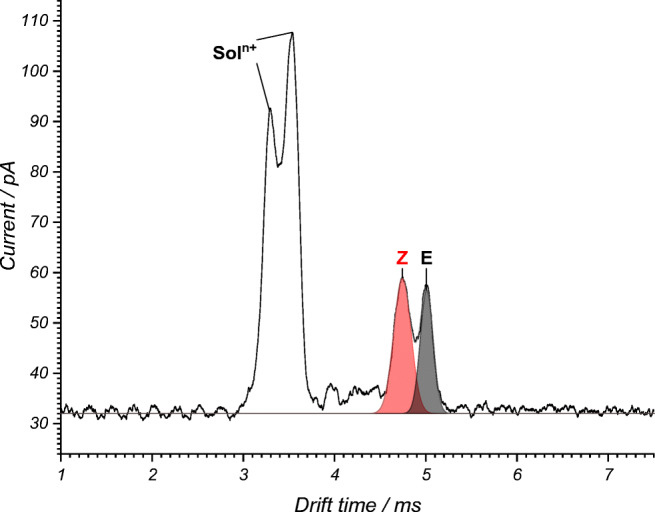
IM spectrum of the *E-* and *Z*-isomer of ethyl cinnamate derivatives ***E*-1** and ***Z*-1** with their drift time maxima at 5.01 ms and 4.74 ms, respectively

In the beginning of the reaction, ethyl cinnamate derivative ***E*-1** was exclusively detected at the outlet of the photoreactor with the light source switched off. After switching on the light source, a peak just in front the *E*-isomer appeared, which can be assigned to the corresponding *Z*-isomer ***Z*-1**. This can be seen in the IM spectrum (Fig. 4), in which both isomer peaks appear. The peak at a drift time of 4.74 ms can be assigned to the *Z*-isomer and the peak at 5.01 ms to the *E*-isomer. From the fraction ratio of the peak areas of both isomeric peaks, the reaction yield can be derived after consideration of the calibration function.

### Calibration

**Fig. 5:**
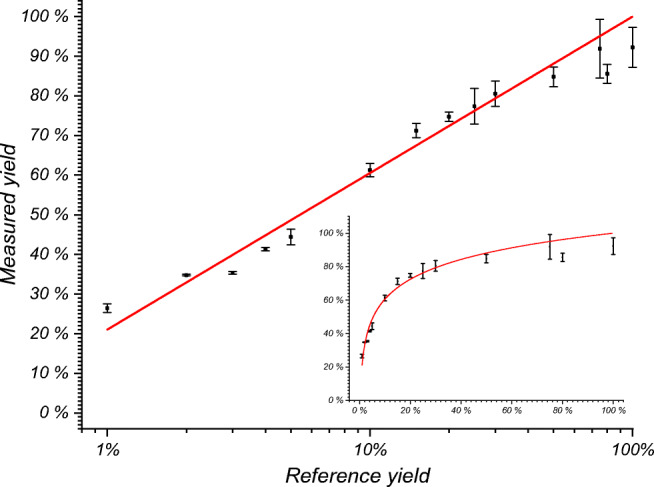
Calibration function for *E*/*Z* isomer mixtures for the IR-MALDI-IMS setup in a semi-logarithmic plot, with *R*^2^ = 0.994, *n* = 14. The inset shows the calibration function for linearly scaled axes. The measured yield is the signal integral ***Z***/(***Z*** + ***E***) in the measured spectrum. The reference yield refers to the actual molar fraction of the *Z*-isomer

While the ionization, and thus the detection efficiency, of different substances varies greatly for ESI or IR-MALDI, and more so for API-MS, no major change is generally expected for isomeric compounds such as the two *E*/*Z* isomers. However, we found that the yields of the photoreactions in the flow reactor obtained by simple peak integration did not follow such a linear trend, and calibration function was recorded in order to correct for this (Fig. 5).

The calibration function (inset in Fig. 5 with linear scaled axes) shows a strong non-linear behavior. It can be concluded that the ionization efficiencies of the *E*-isomer and the *Z*-isomer differ significantly. A significantly larger peak area is observed for the *Z*-isomer than should be expected for the corresponding concentration. Logarithmization resulted in a linearized calibration function (Fig. 5). This correlation allows a sensitive detection of small amounts of the reaction product (*Z*-isomer) and thus the typically rather low reaction yields that are observed in the flow reactor setup according to the short reaction time. In contrast, the calibration function (inset in Fig. 5) for larger yields shows a flatter curve. Consequently, the determination of higher yields is therefore less precise.

**Fig. 6:**
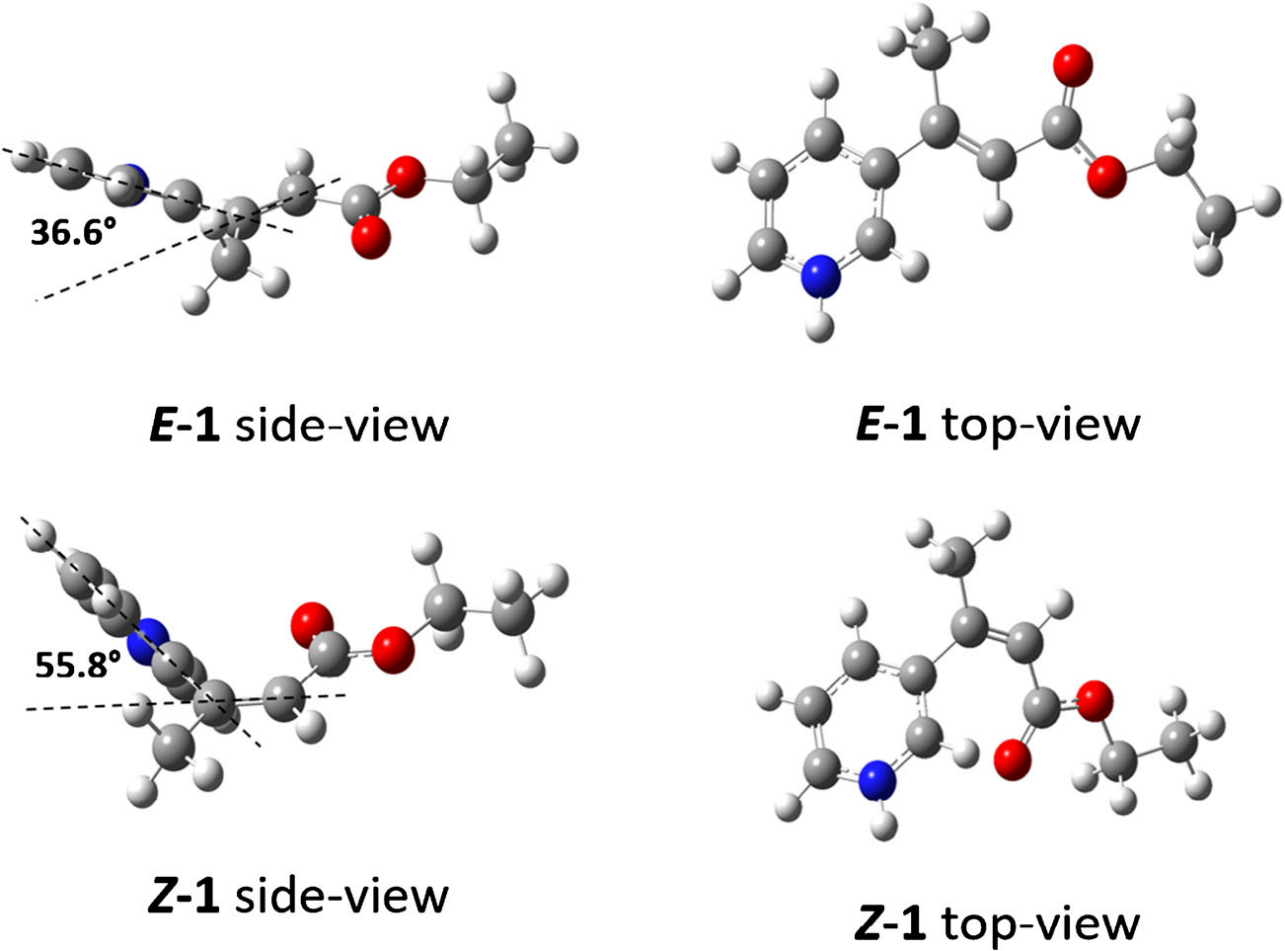
Side and top views of the structures of the *Z*/*E*-isomers, ethyl cinnamate derivatives ***E*-1** and ***Z*-1** (DFT: B3LYP/6-311+G(d,p))

The preferred detection of the *Z*-isomer is a result of a proton transfer reaction from the *E*-isomer to the *Z*-isomer. This protonation can happen either within the nanodroplets (protonation equilibrium) or in the gas phase. The latter, as they occur later, probably determine the protonation ratio. To evaluate the charge transfer equilibrium in the gas phase, the proton affinity of the two isomers was obtained by DFT calculations at the B3LYP/6-311+G(d,p) level. The corresponding energy-optimized structures (side and top views) are shown in Fig. 6. The important structural parameter here is the dihedral angle between the aromatic ring plane and the conjugated double bond. With increasing dihedral angle, the conjugation between aromatic ring and double bond weakens until the conjugation is lost and the π system is restricted to the aromatic ring. This change will affect the proton affinity of the nitrogen atom in the pyridine ring and therefore the proton affinity of the entire molecule. From the *E-* and *Z*-isomers, the dihedral angle increases from 36.6 to 55.8° because it is more energetically favorable, as the steric hindrance in the *Z*-isomer is reduced. This change is associated with an increase in proton affinity from 936.4 to 964.5 kJ/mol for the *E-* and the *Z*-isomers, respectively. This difference in the proton affinity of both isomers may explain the shape of the calibration curve.

### Pressure dependence of charge transfer reactions

**Fig. 7:**
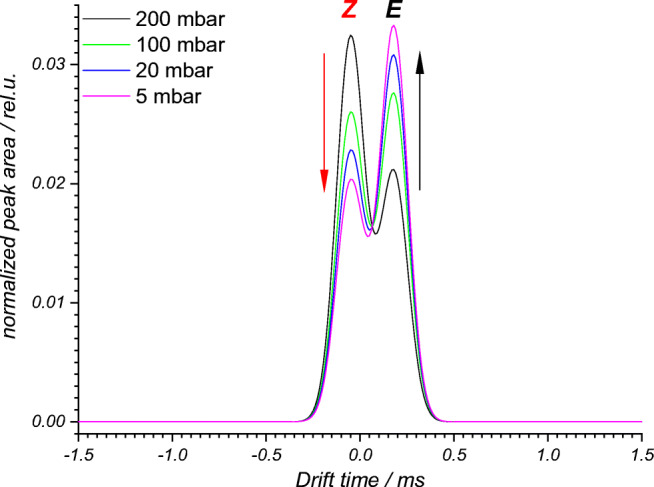
IM peaks for an ***E*-1** and ***Z*-1** 95:5 mol% mixture in dependence of the pressure; for the sake of clarity, only area-normalized and time-scaled (drift-time shifted and peak-width normalized) fit functions are shown

One possibility of suppressing charge transfer reactions was the reduction of pressure, which is associated with a reduction of the collision frequency of the ions in the ionization region and thus the number of reactive collisions. To determine the influence of pressure on the ratio of protonated ions of *Z-* and *E*-isomers, a mixture consisting of 95 mol% *E*-isomer and 5 mol% *Z*-isomer was investigated by IR-MALDI-IM spectrometry in the pressure range between 5 and 200 mbar (Fig. 7). Since the drift voltage was kept constant as the pressure was varied, the ions were detected at lower drift times with decreasing pressure. This reduces the residence time of the ions in the IM spectrometer and thus the number of possible reactive collisions in the surrounding of the transfer capillary.

An essential result of these investigations was the change in the ratio of the peak intensities of the two isomers with varying pressure. Thus, the peak intensity of the *Z*-isomer becomes significantly smaller in relation to the peak intensity of the *E*-isomer with decreasing pressure. The reason is charge (proton) transfer reactions from protonated *E*-isomer to neutral *Z*-isomer due to its higher proton affinity. At atmospheric pressure, a thermodynamic equilibrium between *E*- and *Z*-isomer ions is established due to the high collision frequency. Decreasing the pressure reduces the collision frequency and increases the probability that *E*-isomer ions survive and arrive at the detector.

Although the IM spectrometer should be optimally operated at 5–10 mbar to suppress charge transfer reactions as much as possible, a pressure of 100 mbar was applied in this work. Pressures of 5–10 mbar result in drift times of only a few hundred microseconds. Currently, no amplifiers are commercially available that allow sufficiently fast detection of ions with such drift times, low noise, and amplification factors larger than 1 GV/A. The reduction of charge transfer effects, good sensitivity, and overall performance at 100 mbar were the reason to choose this pressure in the IM spectrometer for the remainder of this work.

The influence of the pressure seemed somewhat surprising, since the dispersion of the liquid occurs at an atmospheric pressure. However, this event took place directly at the inlet of the transfer capillary. The nanodroplets initially formed thus evaporate to a large extent within the heated capillary and in the drift tube of the IM spectrometer, in which the pressure was reduced. This was also where charge transfer reactions took place. With complete suppression of the charge transfer reactions, the ratio of the peak areas should be 5:95, which is not the case. This indicated that a part of the isomer ions was already released at AP or that some transfer reactions still took place in the drift tube at 100 mbar.

### Photoreaction on the microchip

**Fig. 8:**
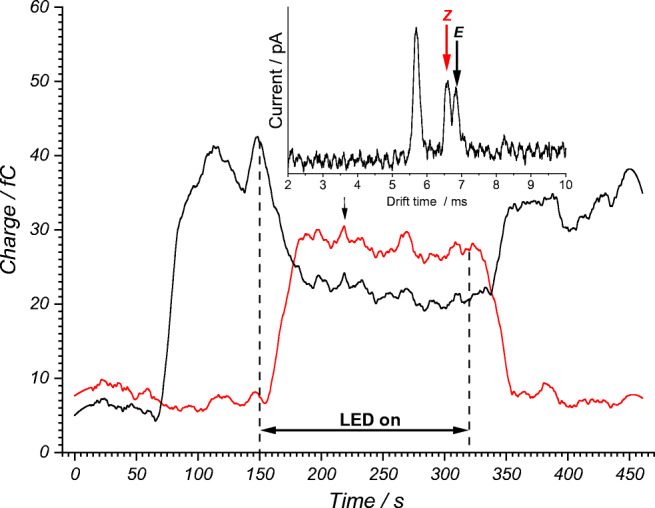
IR-MALDI-IM signals of the *E* isomer ***E*-1** (black) and *Z*-isomer ***Z*-1** (red) recorded over reaction time of the microchip reactor

The time sequence of the experiment is shown in Fig. 8. At time *t* = 0 s, the reaction mixture was injected into the flow reactor. No *E*-isomer is yet detected at the reactor outlet output. At a flow rate of 1 μL/min, an initial *E*-isomer signal was observed after circa 70 s. The LED was switched on at *t* = 150 s to trigger the conversion of the starting material by excitation of the photocatalyst. After a short delay, a continuous decrease of the *E*-isomer signal and an increase of the *Z*-isomer signal were observed. About 30 s after switching on the light source, a stationary state characterized by the maximum conversion of the reaction from *E-* to *Z*-isomers was reached. When the light source was switched off, the concentration of the *Z*-isomer drops and the initial concentration of the *E*-isomer was approximately restored.

The residence time, and thus the irradiation time, of the reaction mixture in the flow reactor was about 70 s, given a sample flow rate of 1 μL/min. Comparable reactions in a batch reactor, as described in the literature, run up 48 h [15]. However, a complete conversion is likely achieved earlier, possibly after several hours. In our exemplary batch reaction from [15], the GC-FID determined yield for the *Z*-isomer was 10% and 96% for riboflavin and 76% and 94% for *fac*-Ir(ppy)_3_ after 2 h and 24 h irradiation time, respectively.

**Fig. 9:**
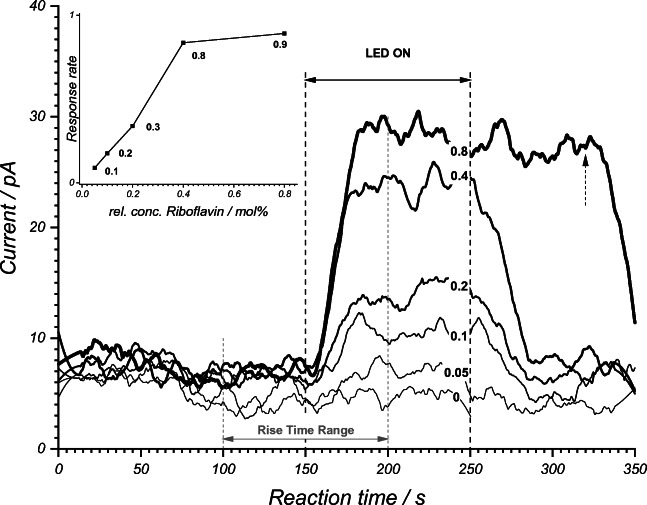
IR-MALDI-IM signal of the *Z*-isomer (product) in dependence of the concentration of the photocatalyst used (0 to 0.8 mol%, from bottom to top with in increasing line width), in this case riboflavin **L**. Inset shows the maximum slope (between 150 and 200 s) and marks the minimum response time of the entire setup at 10 s with a maximum response rate of 0.9 fC/s. For a riboflavin concentration of 0.8 mol%, the irradiation was stopped at 320 s

The influence of the catalyst concentration on the conversion of the *E-* to the *Z*-isomers is shown in Fig. 9. With increasing catalyst concentration (here **L**), an increase of the *Z*-isomer signal was observed in the stationary range. The inset in Fig. 9 shows that the stationary range was reached sooner with increasing catalyst concentration, given the response rate of the system rose from 0.1 to 0.9 fC/s. At a higher catalyst concentration, it seemed that a plateau in the product concentration was reached. The maximum concentration of 2 mol% for the catalysts that was used in this work, resulted from the maximum solubility of the catalysts in the final reaction mixture with MeCN/H_2_O 1:1 (v/v) solvent required for IR-MALDI. This was less of concern for the batch preparations (5 mol%).

### Multiple radiation sources

**Fig. 10:**
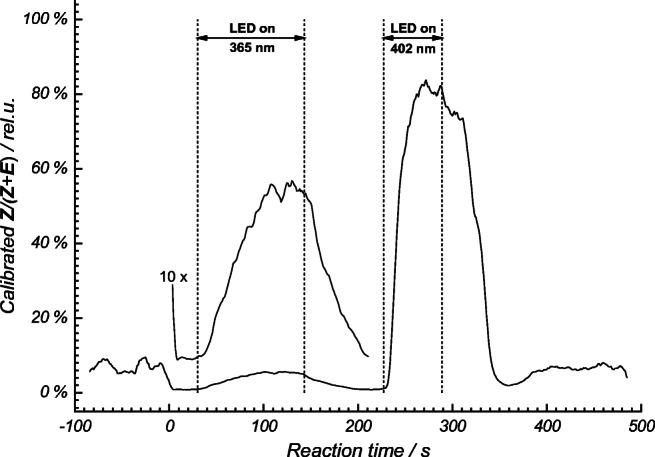
Calibration-corrected yield of the *E*/*Z* isomerization for two different radiation sources, 404 nm and 365 nm sequentially applied to the photoinduced isomerization reaction carried out in the capillary flow reactor (catalyst **M**, [Ir[dF(CF_3_)ppy]_2_(dtbbpy)]PF_6_, 2 mol% in MeCN)

**Fig. 11:**
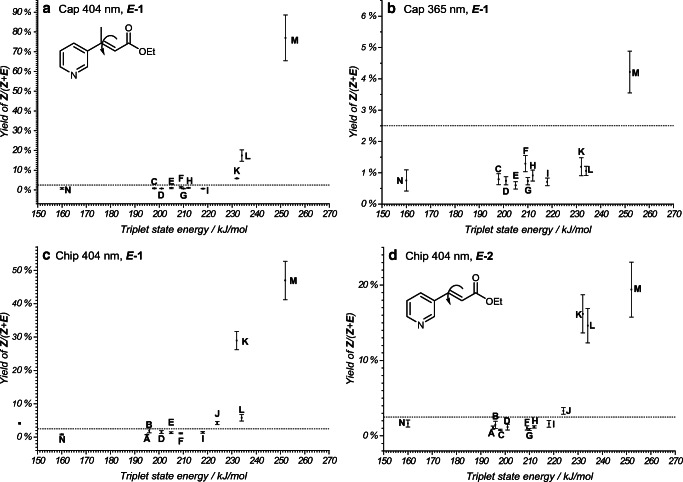
Reaction yields of the *Z*-isomers from its *E*-isomer as starting material: **a** ethyl cinnamate derivative ***E-*1** in the capillary flow reactor at 404 nm excitation (Cap 404 nm, ***E*-1**), **b** ethyl cinnamate derivative ***E-*1** in the capillary flow reactor at 365 nm excitation (Cap 365 nm, ***E*-1**), **c** ethyl cinnamate derivative ***E-*1** in the microchip flow reactor at 404 nm excitation (Chip 404 nm, ***E*-1**), **d** ethyl cinnamate derivative ***E-*2** in the microchip flow reactor at 404 nm excitation (Chip 404 nm, ***E*-2**). Photosensitizing catalysts: **A**, [Ru(bpy)_3_](PF_6_)_2_; **B**, [Ru(phen)_3_]Cl_2_; **C**, Rose Bengal; **D**, [Ru(bpz)_3_](PF_6_)_2_; **E**, Mes-Arc^+^ClO_4_^−^; **F**, 9-fluorenone; **G**, Eosin Y; **H**, [Ir(dtbbpy)(ppy)_2_]PF_6_; **I**, Rhodamine 6G; **J**, 4CzIPN; **K**, *fac*-Ir(ppy)_3_; **L**, Riboflavin; **M**, [Ir[dF(CF_3_)ppy]_2_(dtbbpy)]PF_6_; **N**, [Cu(dap)_2_]Cl. The dashed line at 2.5% represents the threshold of the reaction onset and results from reference measurements, and their standard errors, containing no *Z*-isomer

The spectral overlap integral of the emission of the LED source (*λ* = 404 nm) and the absorption bands of the catalysts may vary greatly. In order to efficiently excite catalysts with varying absorption bands, multiple LEDs may be needed. We implemented an additional UV LED (*λ* = 365 nm) with a 50 times lower intensity (see Fig. S1 in ESM). Still, Fig. 10 shows the product formation for catalyst **M** after sequential switching on and off of the LED light sources with a continuous flow of starting material through the capillary flow reactor. With this setup, that could be extended to even more light sources, we were able to screen the reaction mix with two radiation sources in less than 10 min. With wavelength-tunable light sources in mind, this concept could be extended to a large range of wavelengths. All of which could be continuously screened within one measurement. The shown yields of *Z*-isomer correspond the ratio of ***Z***/(***Z*** + ***E***) after calibration. Due to the lower photon density delivered by the 365-nm LED, a significant conversion could only be obtained for catalyst **M**, Ir(dF(CF_3_)ppy)_2_(dtbbpy)PF_6_ (Fig. 11b). Catalyst **M** was also the most effective catalysts if the 404-nm LED was used (Fig. 11a).

### Product yields

The significantly shorter screening time compared with the several hours in a photo batch reactor makes reaction screening in a capillary or microchip photoreactor superior. In combination with flow injection analysis (FIA), this technique has potential for high-throughput analysis.

In this work, the reaction yield of photoisomerization was determined for a number of different photocatalysts (Table 1). These catalysts were characterized by their corresponding absorption in the UV/Vis range and a wide range of triplet energies. The results of the catalyst screening are shown in Fig. 11 as a representation of the reaction yield vs. the triplet energy of the catalyst. A more precise treatment would consider the kinetics of the energy transfer and *E*/*Z* isomerization.

Herkstroeter and Farid [43] were able to show that the logarithmic rate constants of the energy transfer of different acceptors display a characteristic behavior with respect to the triplet energies of the photocatalysts: from a strong increase in the endothermic regime, a plateau is reached in the exothermic limit [39]. Since analogous measurements were not possible in the experiment described here and, in addition, the kinetics of the overall reaction is more complex, the presentation of the results seen in Fig. 11 was chosen. This representation also allowed direct comparison to results published by Metternich and Gilmour [15].

Metternich and Gilmour have shown a similar correlation for the related substrate ethyl-3-phenylpent-2-enoate in photo batch reactor experiments. This correlation was characterized by a sharp increase in the yield of the *Z*-isomer from a triplet energy of 179 kJ/mol (anthracene as photosensitizer), the formation of a plateau with yields of over 95% (with 2 sensitizers at 60% and 72%), and a drop in yield at a triplet energy of 255 kJ/mol (Fig. 4 in supplementary information of [14]). In that work, a long reaction time of 24 h forced a complete conversion from the *E*-isomer to the *Z*-isomer, thereby effectively masking nuances in the sensitizing efficiency.

In contrast, these small differences in reactivity were more clearly visible in the flow reactors due to the significantly shorter reaction times. Within the scope of a screening, only reaction conversions for the most reactive catalysts were observed. This most reactive catalyst was **M**; however, **K**, **L**, and **J** were above the reaction threshold. Their triplet state energies are in the range of 220–250 kJ/mol. This detection of the most reactive catalysts is of particular importance for synthetic chemists and provides a fast and cost-efficient method for catalyst screening. For these catalysts, a complete conversion after 24 h reaction time was observed by Metternich and Gilmour [15].

The significant decrease of the reaction time in both flow reactors was certainly due to the more homogeneous irradiation of the reaction mixture with an optical layer thickness in the range of less than 75 μm (capillary reactor 75 μm diameter, microchip reactor 20–30 μm channel depth, 100 μm channel width). In contrast, in batch reactors at a high optical density, only a very small volume of the reaction mixture is continuously irradiated. On the other hand, by varying the flow of the starting materials, or even going to stop flow, an extension of the reaction time can be achieved. By doing so, the photoreactions of other catalysts can also be detected.

Figure 11 a and c compare the yields of both photo flow reactors for irradiation at *λ* = 404 nm. In both flow reactors, only the conversions of the reaction under participation of the above-mentioned catalysts were observed, meaning a good qualitative agreement was obtained. While an absolute agreement of the yields cannot be expected due to the different irradiation conditions in both reactors, strong deviations in the relative yields of the catalysts **M** and **L** were observed. Besides the ethyl cinnamate derivate ***E*-1**, a second structurally related *E*-isomer of ethyl-3-(pyridine-3-yl)prop-2-enoate ***E*-2** that is without the methyl group of ethyl cinnamate derivative ***E*-1**, was investigated (Fig. 11d). Comparison of the yields in Fig. 11 c and d reveals a qualitatively similar reaction behavior. The most reactive catalyst was again **M**, with **K**, **L**, and **J** above the threshold. However, yields for *Z*-isomer were lower for this ethyl cinnamate derivative as the missing methyl group leads to a decreased steric constrains in the *Z*-isomer and inferior deconjugation of the π system. Thus, the triplet energy of the formed *Z*-isomer ***Z*-2** is lower than for *Z*-isomer ***Z*-1**, which also allows for back-isomerization of ***Z*-2** to the *E*-isomer ***E*-2** by triplet energy transfer.

## Conclusions and outlook

Using a novel combination of photo flow reactors with an IR-MALDI-IM spectrometer, we found catalyst **M** and also **K**, **L**, and **J** to be effective and efficient sensitizers for the *E*/*Z* isomerization of ethyl cinnamate ***E*-1**. Irradiation time sequences from 10 to 180 s yielded conversion rates of up to 80% and allowed us to identify these four best performing catalysts out of a set of 14 different photocatalysts within only minutes of measurement time for each. When compared with current studies found in the literature, this approach helps to drastically reduce the measurement times to only a few minutes per scan. In addition, given the much smaller volume of the reaction vessel, only 2 nmol (20 μL of a 100 μM solution) of catalyst was used for a single scan compared with 5 μmol of catalyst used in a single reaction with a batch reactor setup. Especially in the context of valuable iridium- and ruthenium-based catalysts, this certainly constitutes an improvement in cost and resource efficiency.

In the future, wavelength-tunable light sources could be used for directly subsequent irradiation periods due to the fast scanning time of the setup. This approach could make wide ranges of wavelengths accessible to reaction performance tests in short timescales. Such an approach would be infeasible in batch reaction–based screening systems.

## Electronic supplementary material

ESM 1 (PDF 1.09 MB)
