# Supplementary material for: In situ monitoring of photocatalyzed isomerization reactions on a microchip flow reactor by IR-MALDI ion mobility spectrometry
Source: Anal Bioanal Chem. 2020 Sep 12;412(28):7899–911. doi: 10.1007/s00216-020-02923-y (PMC7550389; doi:10.1007/s00216-020-02923-y)
Supplement: Supplementary file 1 — (PDF 1.09 MB) [file 216_2020_2923_MOESM1_ESM.pdf]

Analytical and Bioanalytical Chemistry

Electronic Supplementary Material

In situ monitoring of photocatalyzed isomerization reactions on a microchip flow reactor by IR-MALDI ion mobility spectrometry

Chris Prüfert, Raphael David Urban, Tillmann Georg Fischer, José Villatoro, Daniel Riebe, Toralf Beitz, Detlev Belder, Kirsten Zeitler , Hans-Gerd Löhmannsröben

General Methods: Unless otherwise noted, all commercially available compounds were used as received without further purification. Batch reactions were carried out under an atmosphere of dry argon using oven-dried glassware unless otherwise stated. THF was distilled from sodium/benzophenone. Solvents for substrate synthesis and batch experiments were p.a. grade.

Chemicals: All solvents were purchased from MERCK. Purified and filtered water was produced on-site using a Millipore filter system (Quantum TEX, MilliPak Express 40, Millipore). Catalysts were purchased from the following distributors: Eosin y (CAS: 15086-94-9), Rhodamine 6G (CAS: 989-38-8) from Sigma Aldrich; 9-fluorenone (CAS: 486-25-9) from Acros Organics; 9-mesityl-10-methylacridinium perchlorate (Mes-ArcClO_4^- , CAS: 674783-97-2) from TCI, 4,5,6,7-tetrachloro-2′,4′,5′,7′-tetraiodofluorescein disodium salt (Rose Bengal, CAS: 632-69-9) from Alfa Aesar; Riboflavin (CAS: 83-88-5) from Hoffmann La-Roche. Tris(2-phenylpyridinato-C2,N)iridium(III) (*fac*-Ir(ppy)$_3$, CAS: 94928-86-6), tris(2,2′-bipyridine)ruthenium(II) hexafluorophosphate [Ru(bpy)$_3$](PF$_6$)$_2$, CAS: 60804-74-2), 1,2,3,5-tetrakis(carbazol-9-yl)-4,6-dicyanobenzene (4CzIPN, CAS: 1416881-52-1), [Ir[dF(CF$_3$)ppy]$_2$(dtbbpy)]PF$_6$ (CAS: 870987-63-6),), [Ir[dF(CF$_3$)ppy]$_2$(dtbbpy)]PF$_6$ (CAS: 676525-77-2), [Ru(bpz)$_3$](PF$_6$)$_2$ (CAS: 80907-56-8), [Cu(dap)$_2$]Cl (CAS: 1392109-17-9), dichlorotris(1,10-phenanthroline)ruthenium(II) [Ru(phen)$_3$]Cl$_2$ (CAS: 23570-43-6), and all cinnamonic ester derivatives were synthesized in-house.

NMR spectra were recorded on a VARIAN Mercury plus 300 (300 MHz), VARIAN Mercury plus 400 (400 MHz) and BRUKER Avance III HD (400 MHz). Chemicals shifts δ in ppm of ^1H and ^{13}C nuclei were internal references to residual solvent signal ($CDCl_3$: $\delta(^1H)$ = 7.26 ppm, $\delta(^{13}C)$ = 77.16 ppm; DMSO-d_6: $\delta(^1H)$ = 2.50 ppm, $\delta(^{13}C)$ = 39.52 ppm; Acetone-d_6: $\delta(^1H)$ = 2.05 ppm, $\delta(^{13}C)$ = 29.84 ppm and DCM-d_2: $\delta(^1H)$ = 5.32 ppm, $\delta(^{13}C)$ = 53.84 ppm) [1]. Multiplicities of signals are reported as s (singlet), d (doublet), t (triplet), q (quartet), quint (quintet), sept (septet), m (multiplet) and combinations of these multiplicities. Coupling constants J are reported in Hz, rounded to half numbers. All coupling constants J reported refer to J_{HH} couplings if not otherwise stated. Spectra of ^{13}C nuclei were recorded ^1H-decoupled. The applied solvents are specified in the descriptions of the corresponding experiments.

High-resolution ESI-MS spectra were recorded on a BRUKER DALTONICS Esquire 3000 Plus ESI-Ion Trap mass spectrometer or BRUKER DALTONICS Impact II ESI-TOF mass spectrometer with a mass accuracy of 5 ppm. Ionization modes are specified in the descriptions of the corresponding experiments. All reactions were monitored by thin-layer chromatography using MERCK silica gel plates 60 F_{254} (aluminium sheets, 200 µm layer thickness) or MACHERY NAGEL Alugram® Xtra SIL G/UV254 (aluminium sheets, 200 µm layer thickness). Spots were visualized utilizing UV light and/or dyeing reagents solutions of vanillin, ninhydrin, permanganate or molybdophosphoric acids.

GC-FID spectra were recorded on a THERMO SCIENTIFIC Trace 1310 gas chromatograph equipped with a THERMO SCIENTIFIC TG-5MS column (5 % diphenyl- and 95 % dimethylpolysiloxane, 0.25 mm ID, 0.25 µm film thickness, length 30 m) using hydrogen as carrier gas, nitrogen as make-up gas and mesitylene as internal standard to quantify yields.

Irradiation for batch experiments was performed with six EDISON Federal 1W UV LEDs (395 nm) attached to an aluminum heat sink.

Substrate Preparation and Procedures for Batch Experiments

Ethyl (*E*)-3-(pyridin-3-yl)but-2-enoate (*E*-1)

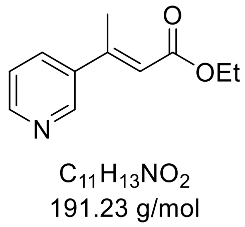

In a 100 mL round-bottom flask, 1.10 g NaH (60% dispersion in mineral oil, 27.5 mmol, 1.1 equiv) were suspended in 50 mL abs. THF. At 0 °C, 6.07 g triethyl phosphonoacetate (97 % purity, 26.3 mmol, 1.05 equiv) were slowly added and the reaction mixture was stirred for 1 h. Then, 3.03 g 3-acetylpyridine (25 mmol, 1.0 equiv) were added. The reaction mixture was stirred for 1 h at 0 °C, was allowed to warm to room temperature and was stirred for additional 22 h. The solvent was removed under reduced pressure and the residue was dissolved in Et_2O and washed with a sat. aq. $NaHCO_3$ solution (100 mL). The aqueous phase was extracted with Et_2O (100 mL) and the combined organic phases were dried over Na_2SO_4. The solvent was removed under reduced pressure. The crude product was purified by distillation (50 cm vigreux column) under reduced pressure (b.p. 106-107 °C, 1.0 mbar) to afford 2.82 g (14.7 mmol, 59 %) of the title compound as a colorless liquid.

^{1}H NMR (400 MHz, $CDCl_3$, 25 °C): δ = 8.73 (d, *J* = 2.5 Hz, 1H), 8.59 (m, 1H), 7.76 (m, 1H), 7.32 (dd, *J* = 8.0 Hz, 5.0 Hz, 1H), 6.14 (m, 1H), 4.23 (q, *J* = 7.0 Hz, 2H), 2.58 (d, J = 1.5 Hz, 3H), 1.32 (t, *J* = 7.0 Hz, 3H).

^{13}C NMR (100 MHz, $CDCl_3$, 25 °C): δ = 166.4, 152.0, 150.0, 147.5, 137.9, 133.9, 123.4, 118.9, 60.3, 17.0, 14.4.

HR MS (ESI+, MeCN): m/z = 192.1017 $[M+H]^+$, calc.: 192.1019.

Ethyl (*Z*)-3-(pyridin-3-yl)but-2-enoate (*Z*-1)

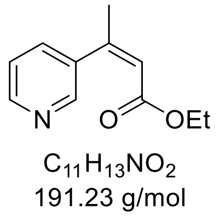

Following Metternich and Gilmour[1], a Schlenk tube was charged with 1.9 mg riboflavin (5.0 µmol, 5 mol %) or 3.3 mg *fac*-Ir(ppy)$_3$ (5.0 µmol, 5 mol%) or 0.9 mg 9-fluorenone (5.0 µmol, 5 mol%) and 1.5 mL MeCN or 1.5 mL *i*PrOH were added, followed by 19 mg ethyl (*E*)-3-(pyridin-3-yl)but-2-enoate (0.10 mmol, 1.0 equiv). The reaction mixture was irradiated with blue LEDs (405 nm) for 24 h. Yields were determined by GC-FID analyses (see Table S1).

Tab. S1 Yields for the photocatalyic isomerization of olefin ***E*-1** in batch reactions. [a] Yields determined by GC-FID analyses.

Entry	Solvent	Photocatalyst	Yield[a] after 2 h irradiation	Yield[a] after 24 h irradiation
1	MeCN	riboflavin	10 %	96 %
2	*i*PrOH	riboflavin	7 %	96 %
3	MeCN	*fac*-Ir(ppy)$_3$	76 %	94 %
4	MeCN	9-fluorenone	20 %	69 %

For another batch experiment, after 24 h of irradiation the solvent was removed under reduced pressure and the crude mixture was purified by flash chromatography on silica gel (hexanes/ethyl acetate; 5-30 % ethyl acetate) to afford 17 mg (89 µmol, 89 %) of the title compound as a colorless liquid.

^{1}H NMR (400 MHz, $CDCl_3$, 25 °C): δ = 8.58 – 8.52 (m, 1H), 8.49 – 8.41 (m, 1H), 7.56 (dt, *J* = 8.0, 2.0 Hz, 1H), 7.32 – 7.27 (m, 1H), 6.02 – 5.98 (m, 1H), 4.01 (q, *J* = 7.0 Hz, 2H), 2.19 (d, *J* = 1.5 Hz, 3H), 1.09 (t, *J* = 7.0 Hz, 3H).

^{13}C NMR (100 MHz, $CDCl_3$, 25 °C): δ = 165.5, 151.7, 148.7, 147.7, 136.8, 134.9, 122.9, 119.7, 60.2, 27.1, 14.1.

HR MS (ESI+, MeCN): m/z = 192.1017 $[M+H]^+$, calc.: 192.1019.

Mechanistic details may be found elsewhere in the literature. [2, 3]

NMR Spectra of Substrates and Products

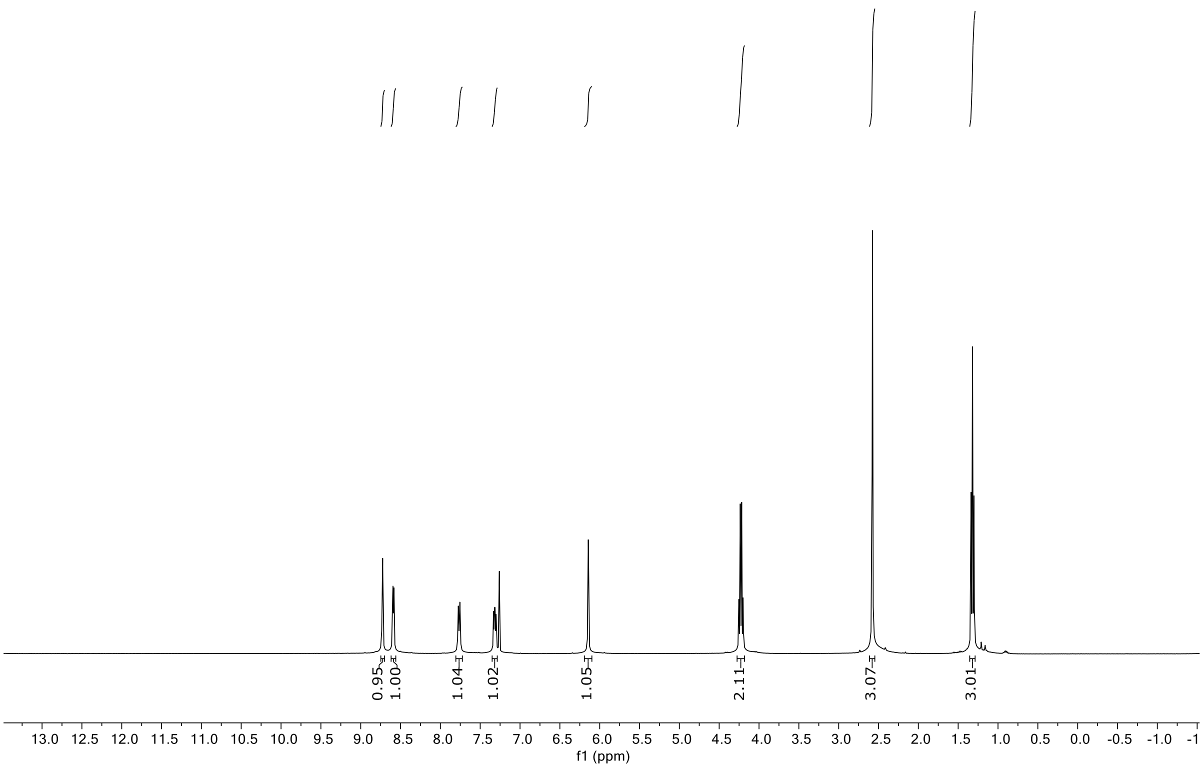

Fig. S1 ^{1}H NMR spectrum of ethyl (*E*)-3-(pyridin-3-yl)but-2-enoate (***E*-1**) in $CDCl_3$

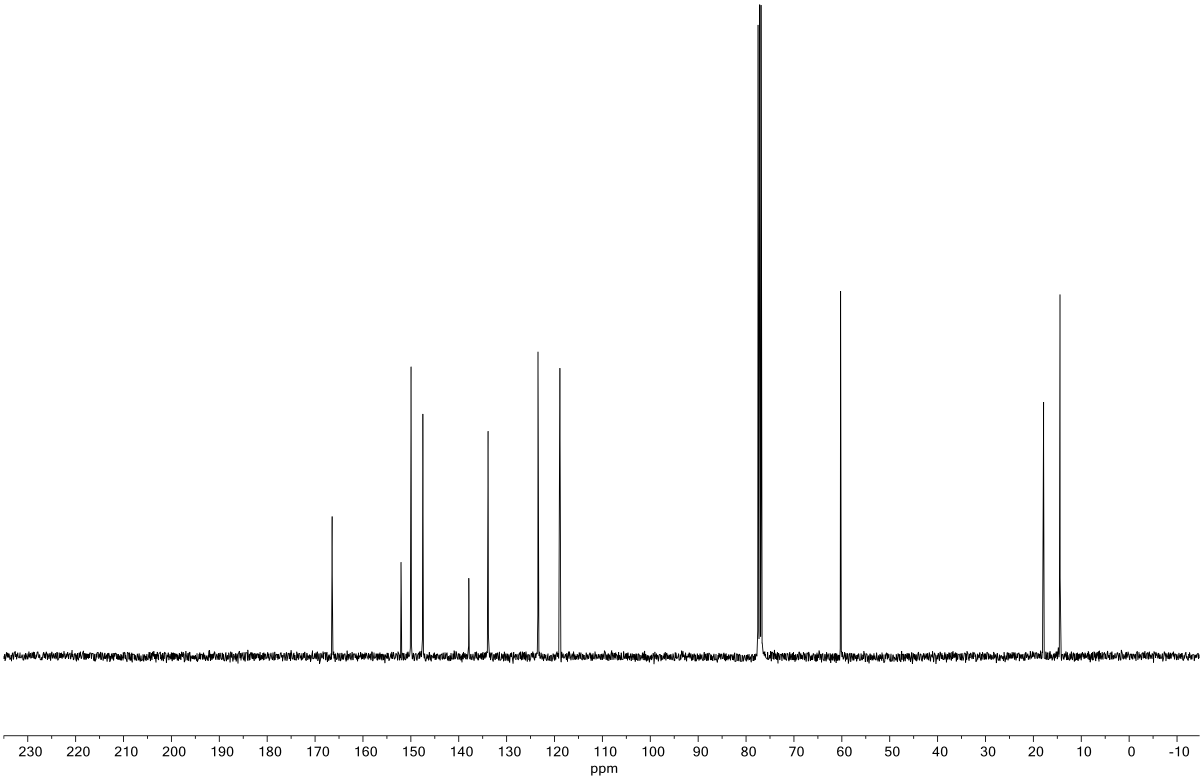

Fig. S2 ^{13}C NMR spectrum of ethyl (*E*)-3-(pyridin-3-yl)but-2-enoate (***E*-1**) in $CDCl_3$

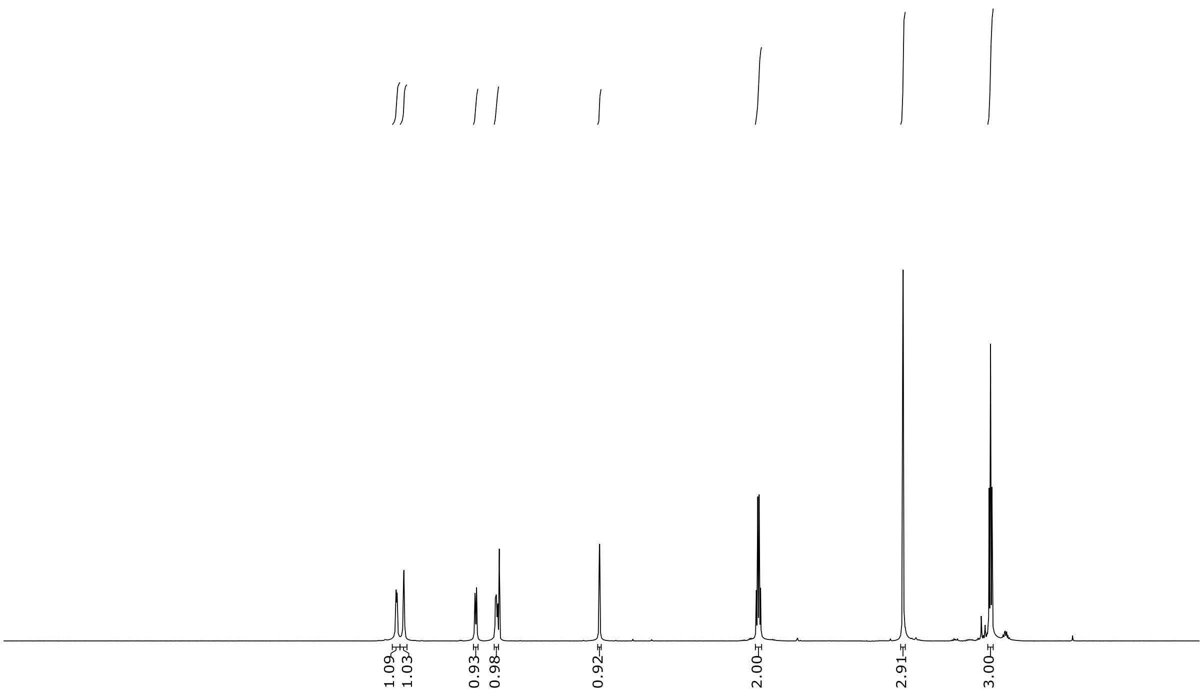

Fig. S3 ^{1}H NMR spectrum of ethyl (*Z*)-3-(pyridin-3-yl)but-2-enoate (***Z*-1**) in $CDCl_3$

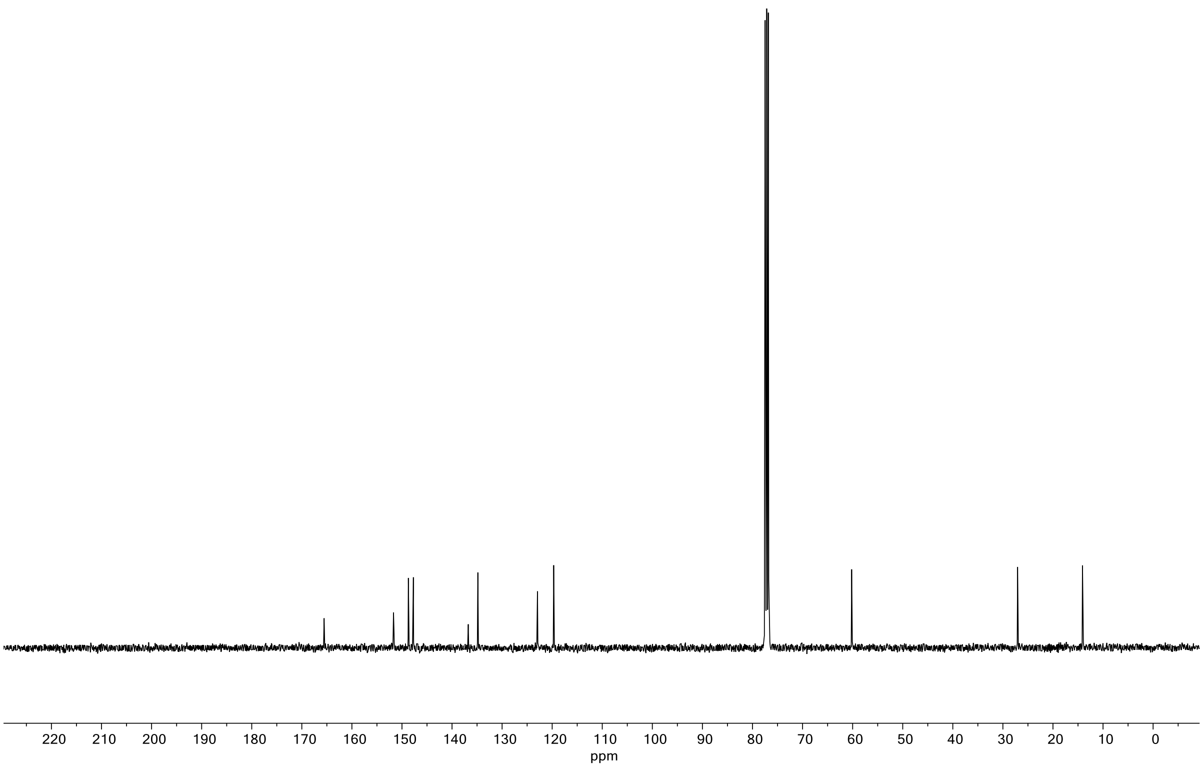

Fig. S4 ^{13}C NMR spectrum of ethyl (*Z*)-3-(pyridin-3-yl)but-2-enoate (***Z*-1**) in $CDCl_3$

Microchip description and fabrication: All chemicals for chip fabrication were bought from Microchemicals GmbH (BW, DEU). Soda-lime microscopic glass slides (76 mm x 26 mm, Carl Roth GmbH + Co. KG, Karlsruhe, BW, DEU) were sputtered with a chromium layer (150 nm, FHR Anlagenbau GmbH, SN, DEU). Foil masks were designed with desired layout by using the vector graphics editor Inkscape (https://inkscape.org/) and printed by DTP-System-Studio GmbH (SN, DEU). After drop casting and spin coating (Spin 120 PTFE, SPS Europe, GE, NL; setting: 4000 rpm, 1000 rpm/s, 30s) of a photoresist (AZ1518) onto the cleaned chrome layers of the microscopic slides and coverage with the photomask, the slides were exposed to the light (14 mJ/cm², 30s) of a mercury arc lamp (Flutbelichter FB5, SÜSS MicroTec AG, BY, DEU). The photoresist was developed by a mixture (1:4 *v/v*) of developer (AZ351B) and water. A patterned chrome layer was obtained by etching the layer by a mixture of perchloric acid solution ($HClO_4$), and ceric ammonium nitrate $(NH_4)_2[Ce(NO_3)_6]$ (TechniEtch Cr01) for 1 minute. Afterwards, the chromium patterned microscopic slide was etched with hydrofluoric acid (HF : NH_4F = 12.5 : 87.5 *v/v*) for 40 min. After neutralizing the HF with a $Ca(OH)_2$ – solution and rinsing with water, the residual photoresist and chrome was removed with acetone and chrome etchant, respectively. Cover slides were manufactured by powder blasting inlet holes into microscopic slides (sandblaster Point 2, Barth Serienapparate, BW, DEU) and bonded to the structured slides running a temperature program (heating ramp to 500 °C in 50 min, maintain Temperature for 15 min, heating to 550 °C within 10 min, maintain temperature for 30 min, heating to reach 620 °C within 15 min, maintain temperature for 180 min, commence cool-down to room temperature within 600 min) in a muffle furnace (muffle furnaces P330, Nabertherm GmbH, NI, DEU). A pulled-glass emitter tip was manufactured at the chip exit, to reduce required flow rate to generate a fluid jet, see Fig. S5.

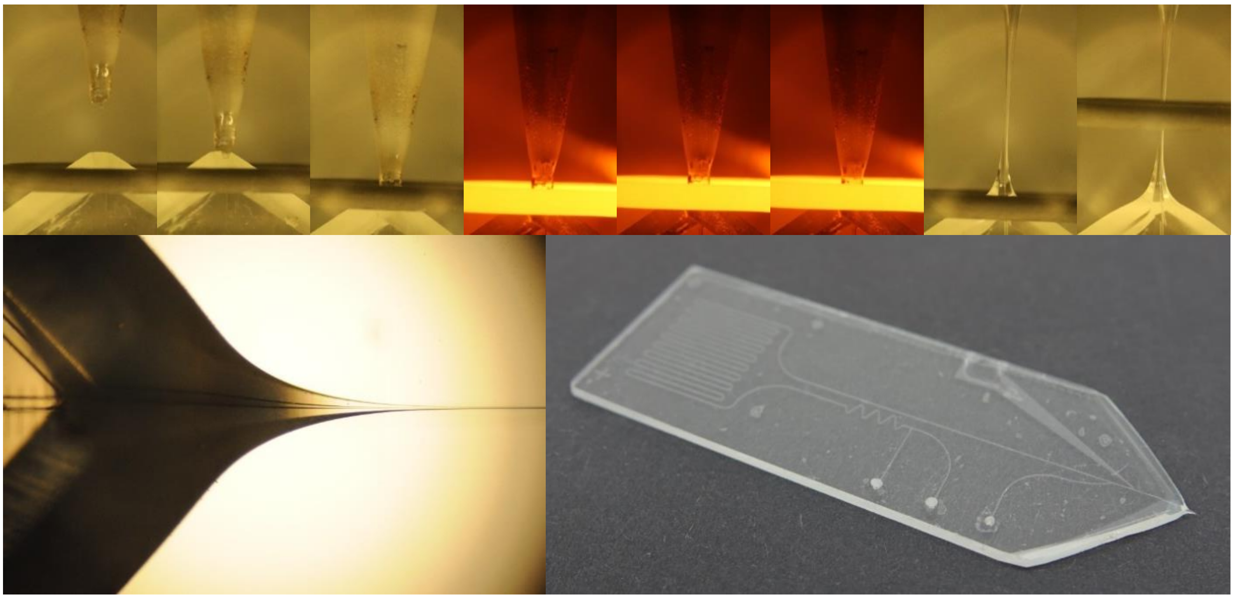

Fig. S5 Top row: Glass emitter pulling process. The chip is at first polished in a V-shape at around a 60° angle at the etched channel, and then positioned in between a wire (Konstantan) with 1 mm diameter. A glass cone is pressed on top of the channel exit, and the wire aligned to the interface of the cone and the chip. While the glass cone is pressed to the chip, both are heated by the current-carrying wire. After melting, the pressure on the glass cone is reduced, and the channel gets pulled by the weight of the glass cone. The emitter is opened simply by breaking the glass fibre at a desired position. Bottom-left: Tip emitter just before breaking off the glass fibre. Bottom right: Microchip used in this study.

Catalyst spectra: The absorption and emission spectra were taken directly successive from the photocatalysts solution (5 µM in MeCN or MeCN/MeOH).

Absorption: UV/Vis/NIR-Absorptions spectrometer Lambda 750 (Perkin Elmer), range: 800 nm - 200 nm, slit width: 2 nm, step width: 1 nm.

Emission: Fluoromax 4P TCSPC (Horiba), excitation wavelength: 402 nm or 365 nm (red vertical line), slit width: 4 nm, step width: 1 nm, integration time: 300 ms, intensity correction for laser intensity fluctuations.

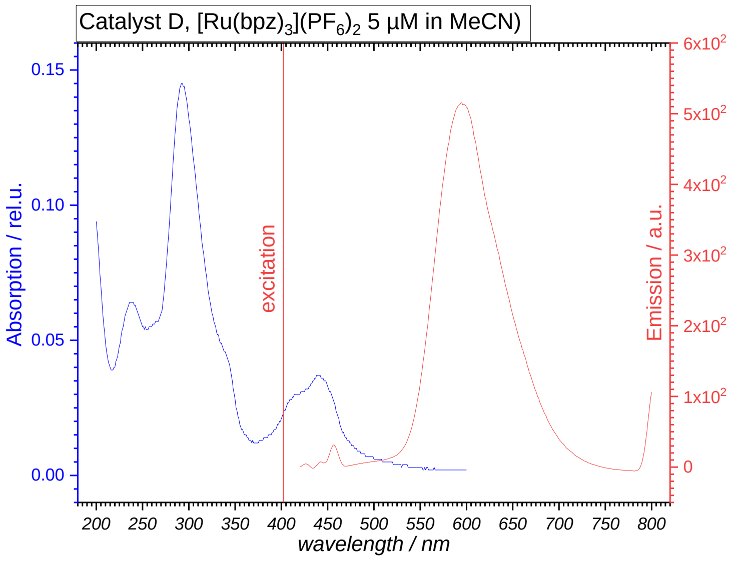

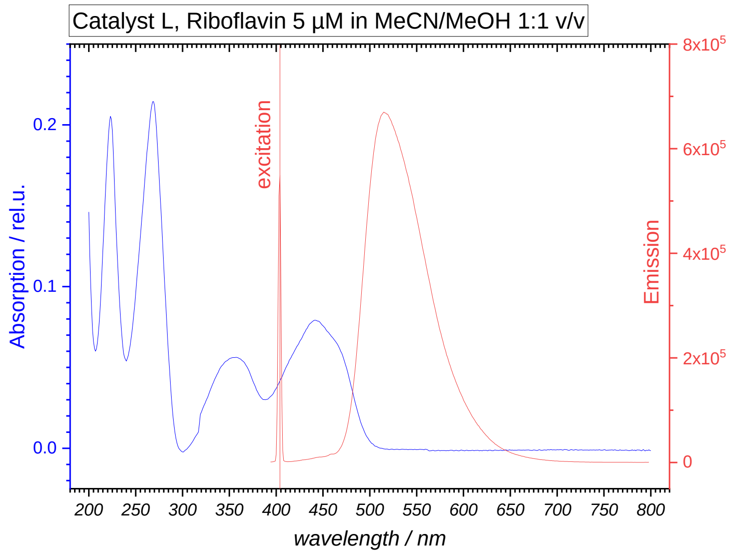

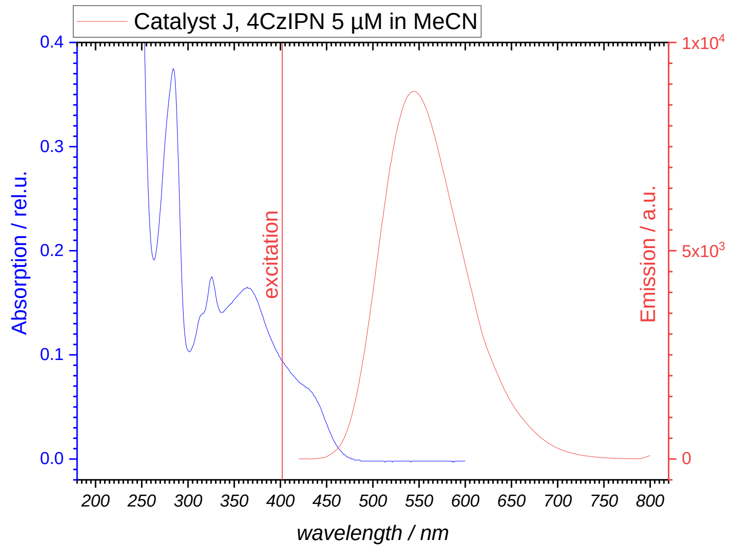

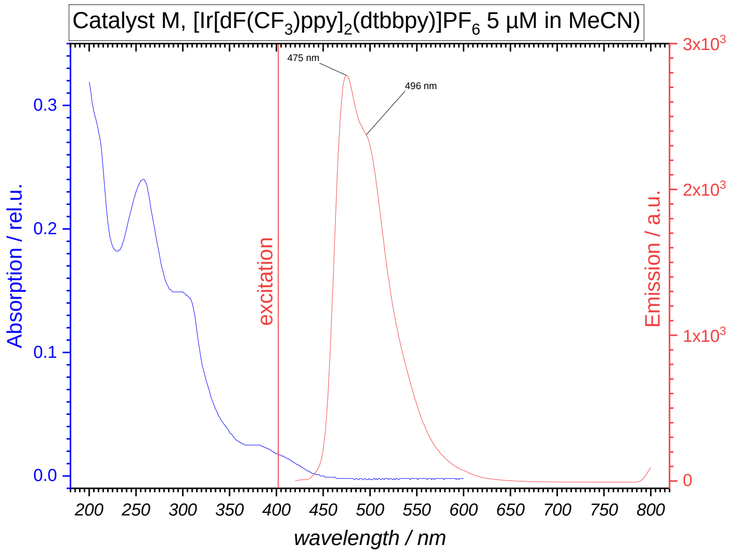

References

1. Fulmer GR, Miller AJM, Sherden NH, Gottlieb HE, Nudelman A, Stoltz BM, et al. NMR Chemical Shifts of Trace Impurities: Common Laboratory Solvents, Organics, and Gases in Deuterated Solvents Relevant to the Organometallic Chemist. Organometallics [Internet]. 2010 May 10;29(9):2176–9. Available from: https://pubs.acs.org/doi/abs/10.1021/om100106e

2. Metternich JB, Gilmour R. A Bio-Inspired, Catalytic E → Z Isomerization of Activated Olefins. J Am Chem Soc. 2015 Sep;137(35):11254–7.

3. Metternich J, Gilmour R. Photocatalytic E → Z Isomerization of Alkenes. Synlett [Internet]. 2016 Oct 11;27(18):2541–52. Available from: http://www.thieme-connect.de/DOI/DOI?10.1055/s-0036-1588621
